# A patient-specific 3D model of the knee to compare the femoral rollback before and after total knee arthroplasty (TKA)

**DOI:** 10.1186/s40634-020-00319-6

**Published:** 2021-01-04

**Authors:** Alexandra Mercader, Timon Röttinger, Amir Bigdeli, Tim C. Lüth, Heinz Röttinger

**Affiliations:** 1 Technical University of Munich, Boltzmannstraße 15, 85748 Garching bei München, Germany; 2 The Munich Center for Arthroplasty, Chirurgisches Klinikum München Süd Am Isarkanal 30, 81379 Munich, Germany

**Keywords:** Total knee Arthroplasty, Mechanism design, Rollback effect

## Abstract

**Purpose:**

Total knee arthroplasty (TKA) is nowadays performed as a standard procedure on a large number of patients suffering from arthrosis. Replacing the knee joint causes changes in the geometry and kinematics of the knee, which are unique to each individual. This research focuses on the method to detect these changes after TKA and on the impact on the knee movement. This approach could reduce complications in patients with post-operative pain and reduce the number of revisions.

**Methods:**

A 3D model of a patient’s knee was made by measuring the movement with a medically certified infrared stereo camera. This measurement was combined with the 3D model of the patient’s bones, previously segmented from the CT scan. This model is printed in 3D, one part being the mechanism that follows the movement of the patient, and the other part being the 3D copy of the femur and tibia bones. The knee replacement operation is performed directly on the model and the resulting rollback is being measured before and after TKA.

**Results:**

We observe a difference in the rollback before and after TKA on the 3D printed model. The variation in size and shape of the femoral implant compared to the natural femur condyles is one of the reasons for the changes in the rollback effect. The rollback is half as large after the prosthesis insertion, which confirms the fact that the femoral prosthesis geometry influences the knee kinematics.

**Conclusions:**

In this study, a first 3D model combining the patient-specific kinematic and the geometry of his bones has been constructed. This model allows the surgeon to validate the plan of the operation, but also to understand the problems and consequences generated by the prosthesis insertion. The rollback is one of the most important motion of the knee joint and this behavior could be quantified, providing comparative analysis of the knee joint before and after the operation. As a future study, the model could be used to analyse more parameters of the TKA such as the impact of different implantation methods.

**Supplementary Information:**

The online version contains supplementary material available at 10.1186/s40634-020-00319-6.

## Background

Modern knee arthroplasty proves to be a successful therapy to remedy the pain and functional restrictions associated with osteoarthritic knees. Despite the positive re- sults of knee arthroplasty [35], a consistently high proportion of approximately 20% of patients is not satisfied with an implanted artificial joint [7, 37, 38]. The reasons for these persistent poor results are multifactorial but seem to be mainly related to a malalignment of the implanted artificial components [2]. This mismatching can be caused by the prosthesis design and by the implant positioning or even by both [7]. Routinely preoperative planning in a coronary and sagittal plane limits the im- plant sizes that are likely to be used and provides information on the alignment of the components to reconstruct a correct coronal axis. Still, there are no indica- tions of the biomechanical effects. Former and modern implant designs, including the custom-made products, do not replace the resected bone one-to-one and thus change the biomechanics of the knee joint per se. Also, essential biomechanically relevant structures, such as the anterior cruciate ligament and often even the posterior cruciate ligament, are resected by implantation of an artificial knee joint. The resection of the ligaments can cause biomechanical changes that are have not yet been quantified in the literature.

Various biomechanical concepts for implanting the artificial knee joints are dis- cussed today, such as mechanical alignment (MA), anatomical alignment (AA), adjusted anatomical alignment (AAA), kinematic alignment (KA), restricted kine- matic alignment (RKA), gap balanced alignment (GBA) and measured resection (MR) [3, 10, 12, 14, 29, 30, 33]. The inconsistent results of the different biomechanical alignments are lighting a controversial discussion. So far, none of these concepts has proven convincingly superior to the others.

Even progressive adaptations of the prosthesis design to the natural conditions do not lead to a reliable reconstruction of the preoperative biomechanics in the postoperative result. For example, ambitious prosthesis concepts try to maintain the essential structures of the biomechanical coupling of a knee joint via a bi-cruciate retaining TKA. However, a reconstruction of the pre-existing natural biomechanics of the knee joint has so far not been successful either [5].

An interesting study has shown the influence of the prosthesis on the rollback [19]. Restoring the rollback as it was before the operation leads to better postoperative results and ensures that the prosthesis does not impair the overall mobility of the knee [18]. These studies by the same author prove that, on a computerized model, regardless of the surgical technique, the rollback is reduced after TKA. This is already an interesting change that has not yet been tested on a specific patient’s knee. In addition, performing the TKA on a computer is a challenge for the surgeon and errors can occur in the positioning of the implant.

Further controversial discussions deal with the use of high-tech aids such as navi- gation, robotics, and patient-specific instrumentation (PSI) [8, 10, 30]. So far, these costly techniques have not been able to contribute to a generally recognized im- provement in results.

The fact that each individual knee joint is unique in terms of the overall complexity of size, shape, special biomechanical features, ligament guidance, muscular control, and functionality makes the desire for a general understanding of detailed knee biomechanics very difficult. Knees from cadavers are essential for understanding the joint and help to explain and reproduce the replacement operation on a healthy knee. They bring knowledge about the different techniques used to replace the surface of the bones. The surgery is performed after the death of the patient and is a one-time operation for each cadaver. The values determined in the laboratory utilizing tests on cadaver knees can, therefore, only be transferred to a specific knee joint to a limited extent and are not suitable for comparing simultaneously the results before and after the operation. Moreover, the possible pain due to the resulted implantation can not be evaluated [21, 39].

Computer models also help to understand the knee kinematics. However, these models are made from an average knee and therefore may represent a large part of the population, but they do not represent a specific case of a patient with pain. Following Pianigiani et al. [31], the knee model do not provide a three-dimensional understanding of the effects of the prosthesis insertion due to the complexity of the knee movement and the restrictions of the computer screen. Computer models can be used to analyze the pressure in the knee joint over the entire surface of the femur and tibia bones, as well as the tension and stress of the materials, but provides only information about eventual fractures, and not about kinematic malalignment [36].

**Fig. 1:**
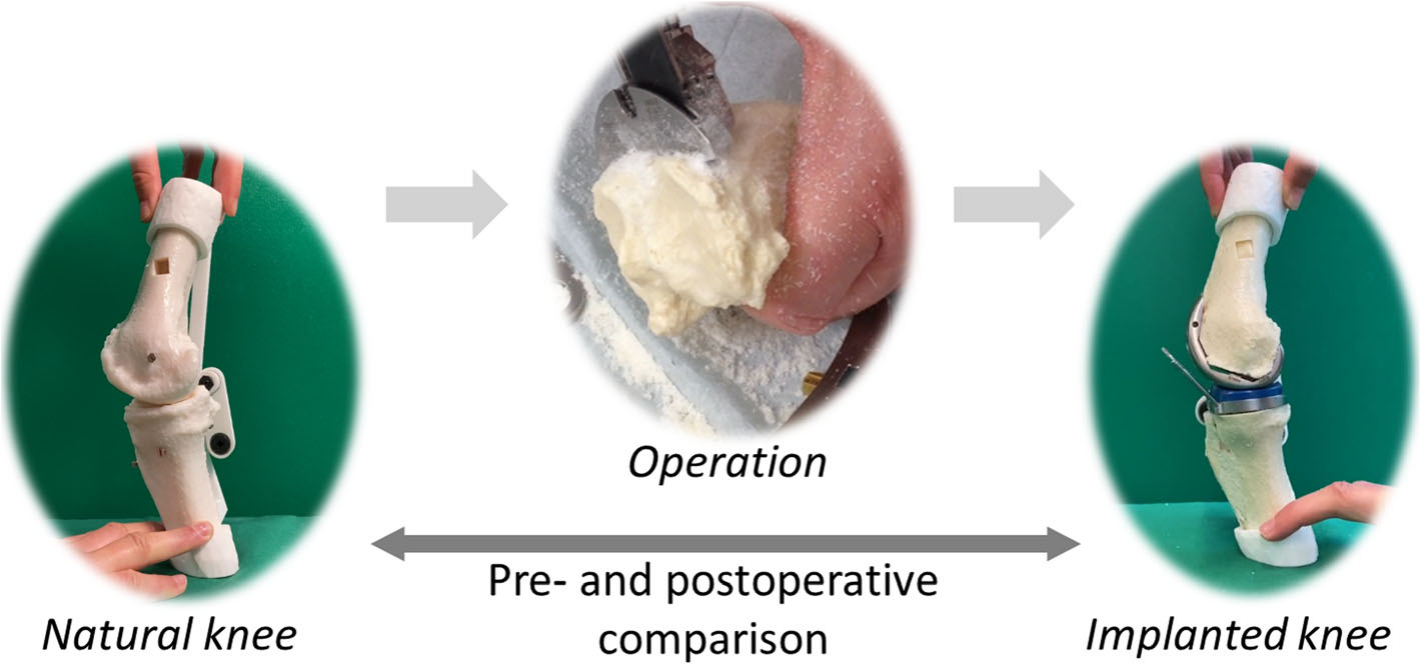
Illustration of the proposed 3D model to compare pre- and postoperative kinematics and behavior of the knee

It is, therefore, desirable to develop a real 3D model that makes the functional effects of different alignments of the implants visible in the specific individual case. The model described below allows a kinematic comparison of a knee joint after prosthesis implantation with the opposite side, but also to observe preoperative kinematics and detect possible issues during the operation process (detailed in Fig. 1). The individualized model can also be used to assess the effects of changed implant positions, and be used to validate the study made by Koh et al. [19]. In case of malfunctions after implantation of a knee endoprosthesis, the model allows to analyze existing conflicts. By comparison with the opposite side or with a preoperative situation, disorders become visible. The following section deals with the creation of the movable 3-D model in a specific, individualized case, here the simulated implantation of a prosthesis during a” Measured Resection”. In the experimental section, a first cutting test is carried out to show the advantages of using such a kinematic model for the validation of the prosthesis placement. In the results, the femoral rollback is compared before and after the operation on the model, which should confirm the hypothesis that implantation has an impact on specific knee kinematic parameters such as rollback.

## Materials and methods

### Data acquisition

**Fig. 2:**
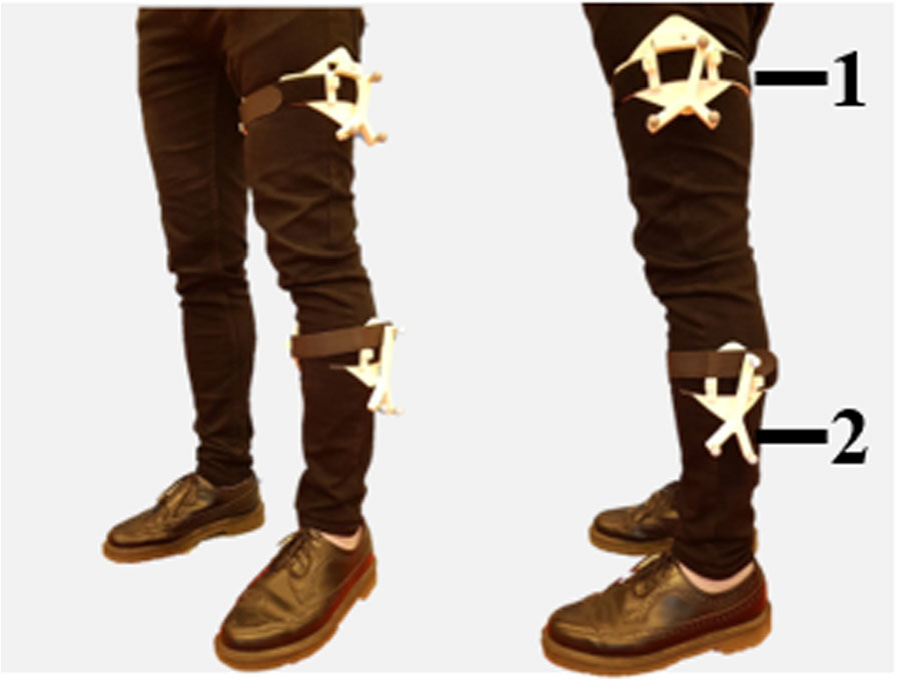
Sample figure title. Figure legend text

The motion of the patient’s knee is recorded to create the patient’s specific model. To accurately measure knee movement, a reliable method reducing skin movement artefacts due to the subcutaneous muscles is required. For the measurements, an infrared stereo camera (Polaris Vicra, Northern Digitial Inc., USA) is used to identify the position of markers placed on the upper and lower leg. The main error that can happen during the measurement is due to the movement of the markers in respect to the bones. Many studies have already tried to solve this issue and the most successful method is to anchor the markers on the bones [32]. However, as this method is too invasive, the choice remains between placing the markers on the skin or on a platform attached to the leg. According to Garling [13], when the marker is mounted on a plate, the measurement is more accurate than when the marker is attached to an elastic band. The measurement is therefore carried out in this research using shells installed on the upper and lower legs. These shells fit all sizes of thighs, 3D printed and padded with a layer of neoprene to prevent slipping along the leg. They are fastened by straps to adjust the pressure of the shell on the limbs. The cylindrical shape of the shell ensures that the shell is firmly attached to the leg and that the measured tracker is placed on the surface of a cylinder around the bone. The trackers 1 and 2 are defined as in Fig. 2.

**Fig. 3:**
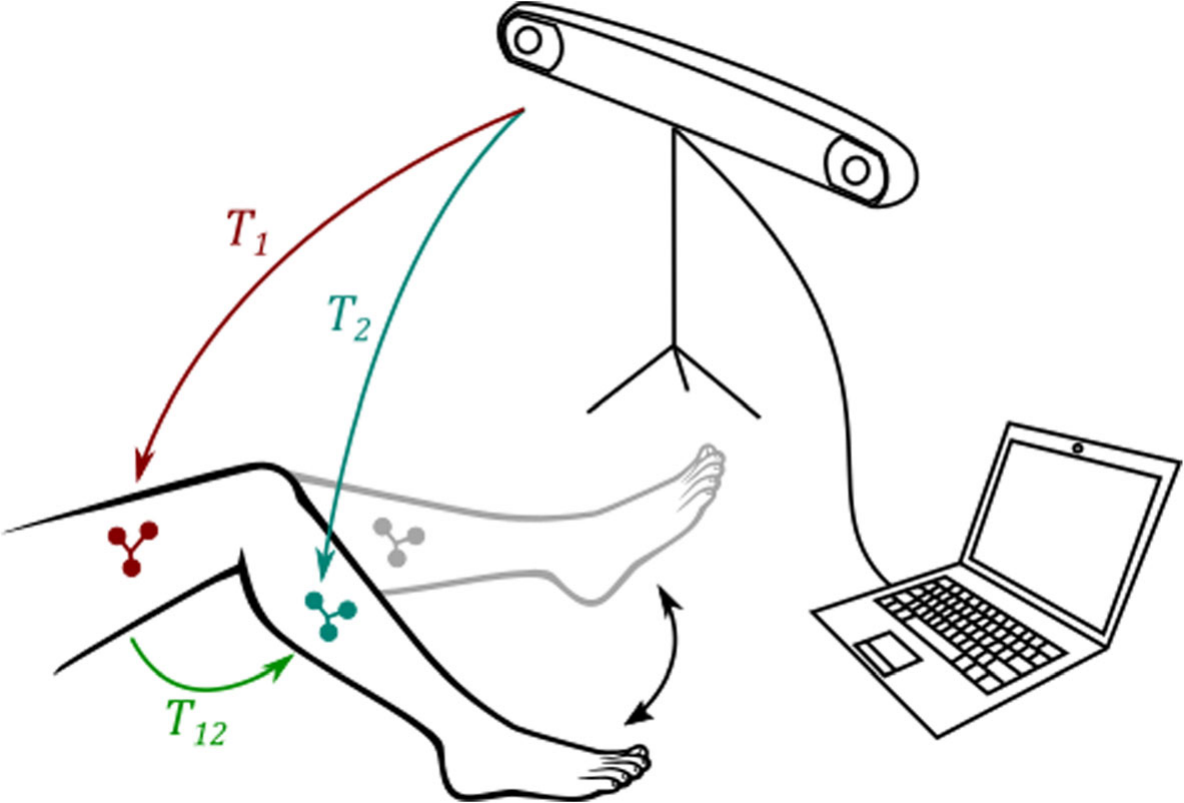
Measurement setup with the stereo camera, the computer and the patient moving his leg without touching the floor

**Fig. 4:**
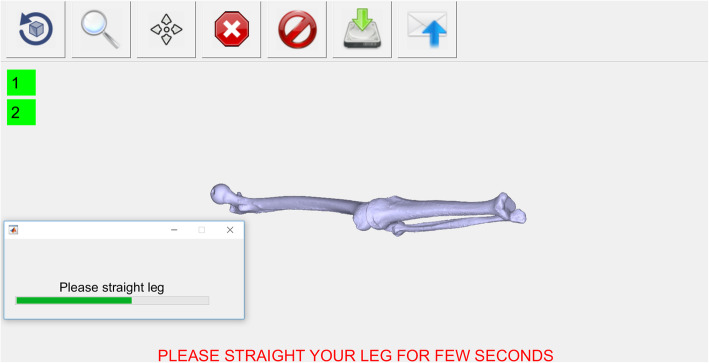
Measuring the straight leg position with the implemented medical software

To assist the doctor’s task, a fully automated user interface programmed with Matlab (MathWorks, Natick, USA) has been implemented for the measurement of the patient’s motion. The complete setup of the camera, the computer and the patient is illustrated in Fig. 3. The software guides the physician to properly position the camera in front of the patient, and saves the camera measurements on the computer. During the measurement, the muscles should not be activated by any additional external force, as this creates muscle contractions and suddenly creates a shift in the measurements. While recording, the patient is sitting on a high chair, with the foot off the floor, and performs several knee flexions and extensions without touching any surrounding objects with his leg. The extended leg position is also recorded (Fig. 4) as the zero angle reference to display the leg model in real time.

### 3D model of the knee

**Fig. 5:**
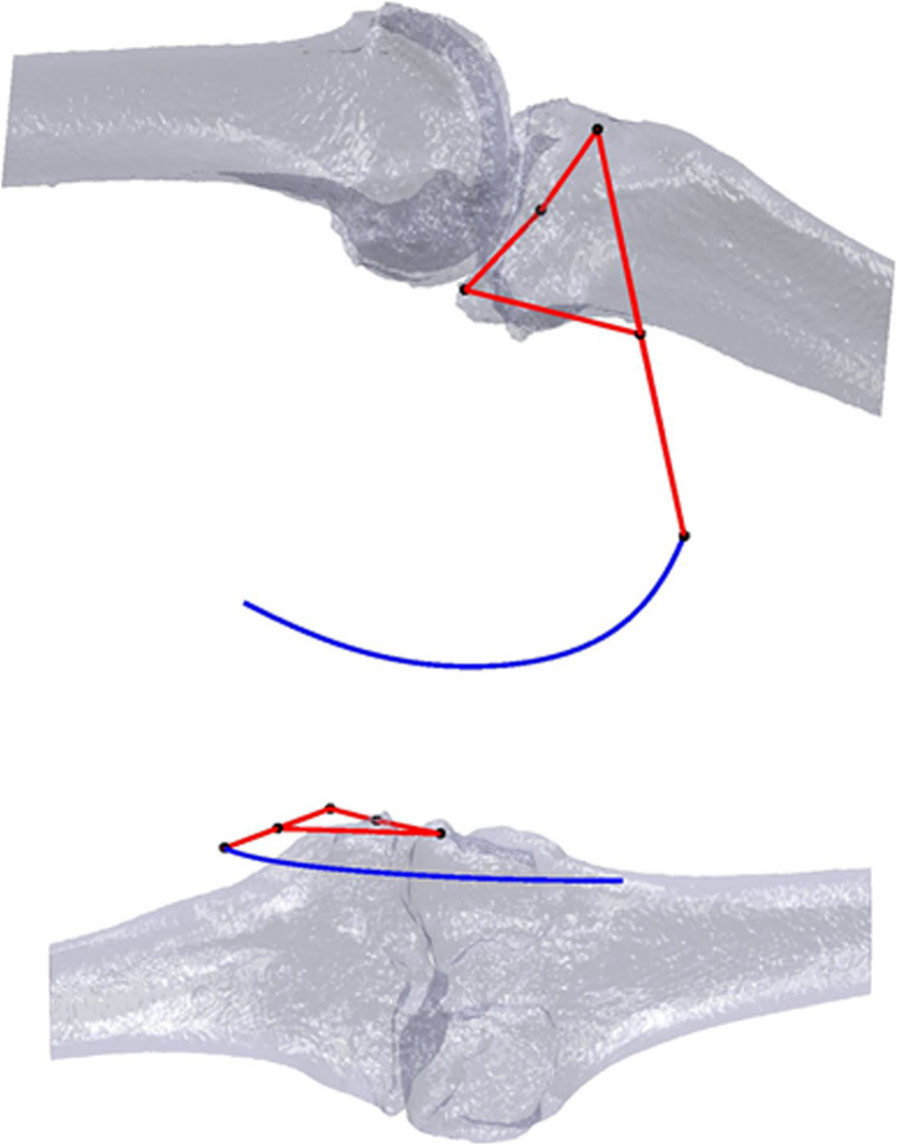
Illustration of the computed four-bar mechanism (red) for the patient’s 3D knee model (grey) in the plane of the measured tibia’s positions, with the trajectory of the coupler point of the four-bar (blue)

**Fig. 6:**
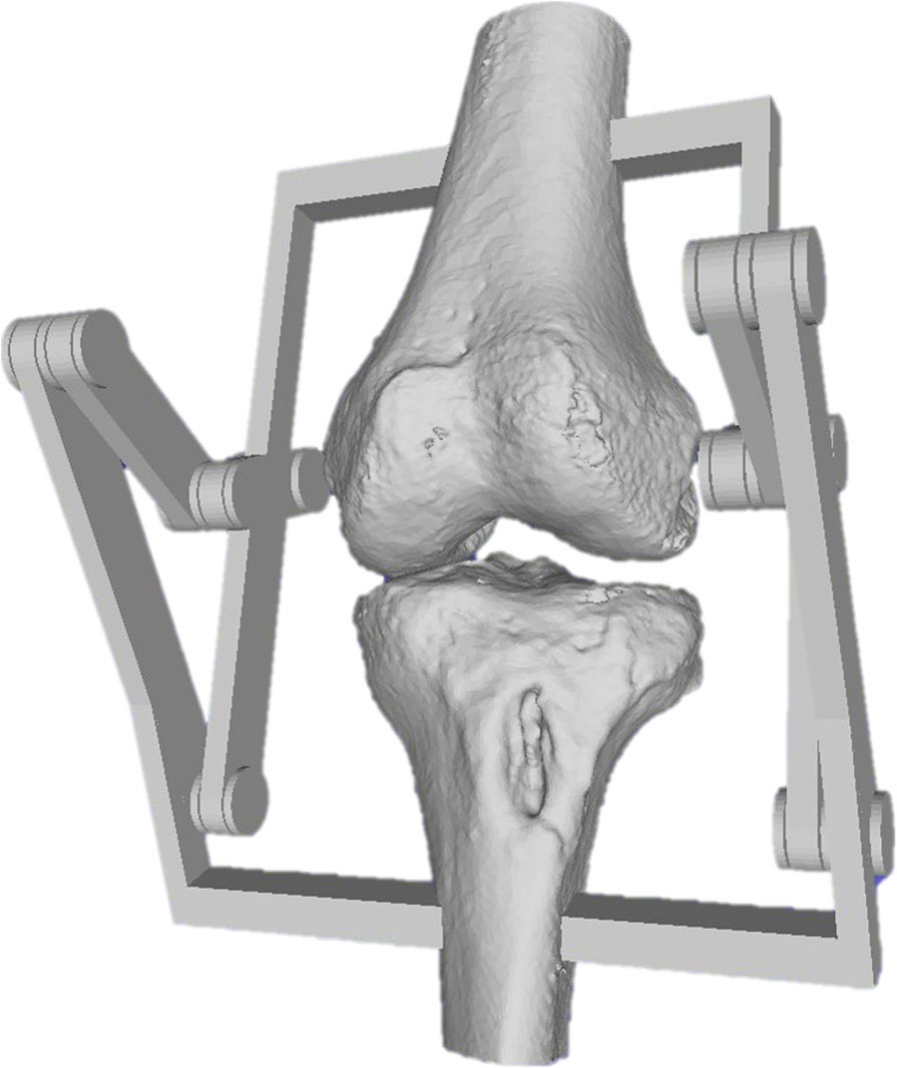
3D knee model with the four-bar mechanism constructed in 3D for a single piece printing process

A CT scan of a patient’s knee is retrospectively used to make a 3D model of the femur and tibia bones. Both bones were segmented by a radiologist using MIMICS software. The CT volume has a dimension of 512x512x431 for an accuracy of 0.7383 × 0.7383 × 0.5 mm. In this study, only the tibiofemoral joint is studied, to check the influence of the prosthesis’ position on the femoral rollback. The extended po- sition of the patient’s knee measured with the system is equivalent to the position during the CT scan, and is therefore used to place the bones relative to the trackers in this recorded position. The movement of the bones is ensured in 3D with centimeter accuracy relatively to the real movement of the patient’s bones with a non-invasive method [26] thanks to the infrared recording technique of the stereo camera (Vicra). The design of the mechanism is performed by optimization so that the trajectory of the planar four-bar mechanism fits the 3D trajectory of the knee similarly to [24, 25]. The four-bar mechanism was specially chosen because the cruciate ligaments are assimilated in literature to such a mechanism according to Nietert [28] and Menschik [23]. As a result, the obtained planar four-bar linkage guides the tibia along the interpolated trajectory. In Fig. 5, the four-bar in red lies in the computed main plane from measurements. The blue curve is the interpo- lated position of the center of the tracker for the whole knee flexion. The four-bar mechanism is equivalent to the cruciate ligaments but holds the bones outside of the knee joint. This is an advantage because the surgeon can insert the prosthe- sis without damaging the four-bar mechanism and therefore the movement of the bones. The resulting bone model with the constructed four-bar linkage is shown in Fig. 6. The four-bar linkage is already assembled to print the knee together with the four-bar in one piece, which does not alter the mathematically computed position of the mechanism relatively to the bones.

### Experiment

The knee replacement is made without the four-bar mechanism and thus without changing the kinematics of the joint (Fig. 8). The goal of the experiment is to show the influence of the constraints caused by the ligaments on the prosthesis’ position.

If the prosthesis is not correctly positioned, the movement will be blocked or will show a lift-off and the flexion may not proceed in the natural way. The hypothesis is formulated in terms of the rollback, one of the most frequently discussed parameters, which is then, in the case of a knee replacement operation according to a surgeon’s routine, modified.

The experiment should show that the prosthesis’ geometry affects the movement of the knee joint. The restriction made in this research by using a four-bar linkage to guide the bones helps to focus on the influence of the geometry over the joint kinematics. For the validation of the hypothesis, one form and one orientation is being tested to focus on the influence of the prosthesis and compare a mechanical parameter before and after the surgery.

**Fig. 7:**
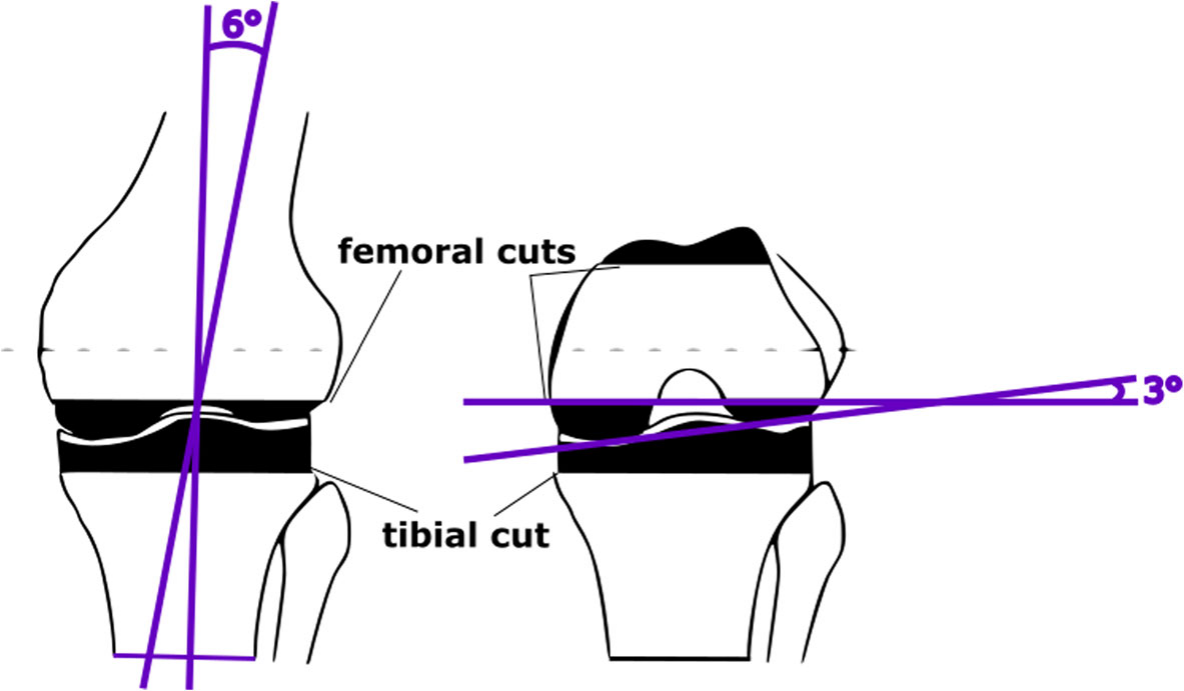
Illustration of the measured resection of 6 degrees of valgus and 3 degrees of external rotation

The model allows the application of all common biomechanical concepts for the implantation of a TKA. In this described case, the TC PLUS PRIMARY (Smith & Nephew) prosthesis is implanted following the measured resection steps under the orientation of the transepicondylar axis (TEA), the anterioposterior axis (AP) and the posterior condylar axis (PCA) such as explained in [1]. The measured resection alignment tries to restore a neutral hip–knee-angle axis. With intramedullary instrumentation, a six degree femoral component angle, and neutral tibial component angle in the coronal plane, a neutral femoral component flexion relative to the sagittal mechanical axis and six degrees of the posterior tibial slope were aimed. The femoral component rotation was determined under orientation to the surgical femoral epicondylar axis. The angles of 6 degrees of valgus and 3 degrees of external rotation as cut on the model are illustrated in Fig. 7.

**Fig. 8:**
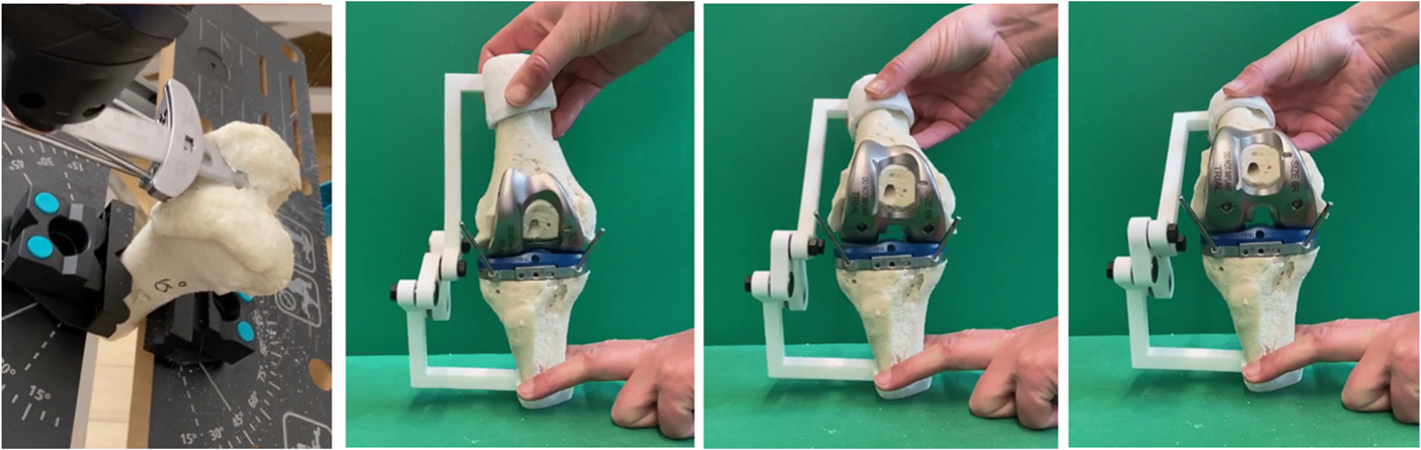
Different steps of the total knee replacement surgery on the patient specific model, from left to right: femur cut, tibia cut, 0, 30 and 60 degrees of flexion after surgery

The model is cut according to the different surgical steps of total knee replace- ment surgery. Figure 8 shows the different cutting steps of the surgery. The cutting process is made with orthopaedic saw blades, as for a real operation. The femur and tibia models are made of polyurethane, independently from the mechanism, so that the bone surface can be accurately cut without being hindered by the constraints of the mechanism. The mechanism is printed in 3D, in PA2200, and the femur and tibia bones can be attached directly to the mechanism after the prosthesis insertion is completed. After femur and tibia are being cut, the surgeon installs the prosthesis according to the plan and connects the bones to the mechanism. The resulting knee model is shown in Fig. 8. Thanks to this model, the surgeon can elaborate a surgery plan for a specific patient and execute it on the corresponding 3D kinematic knee model of the patient. With the external four-bar mechanism, the surgeon can analyze the postoperative effects on the model without modifying the preoperative kinematics. For the observation, the bones are moved according to the preoperatively measured flexion thanks to the mechanism. The operated knee is shown in Fig. 8 for flexion angles of 0, 30 and 90 degrees.

**Fig. 9:**
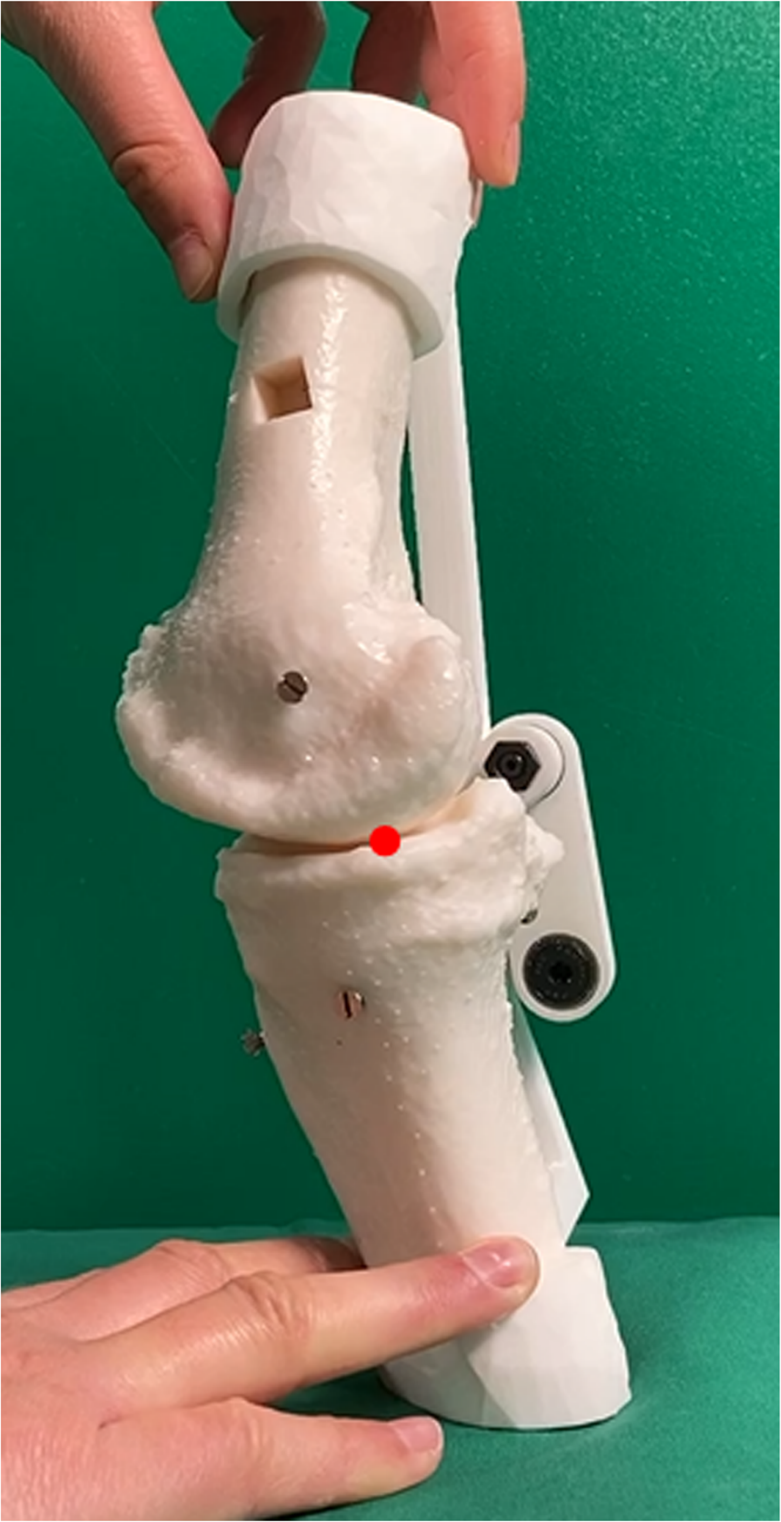
Knee model before surgery, shown in the sagittal plane with the measured contact point (in red) between femur and tibia

**Fig. 10:**
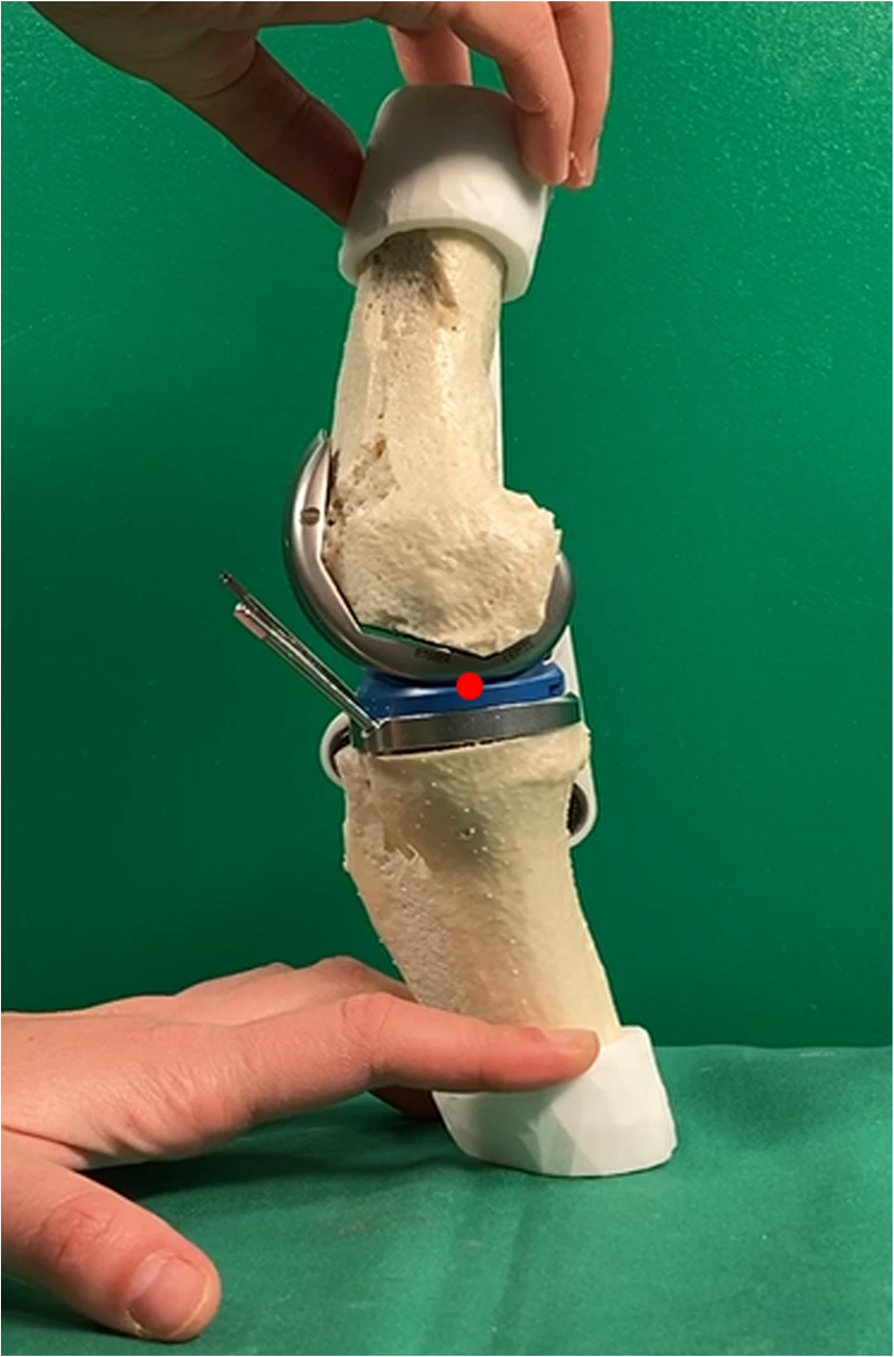
Knee model after surgery, shown in the sagittal plane with the measured contact point (in red) between femur and tibia

The rollback motion is being measured by observing in the sagittal plane the displacement of the contact point between the femur and the tibia. The measured values are normalized by taking 0 mm as the value in the extended position. The value corresponding to 0 mm is taken as the centre of the tibial plateau for both models, before and after the operation. Figures 9 and 10 indicate the point of contact measured during knee flexion. This contact point is continuously evaluated from the video recorded in the same sagittal plane and is compared with the contact point on the same model without implant. For better accuracy, the mean of three measured flexions is taken as final value. The rollback is measured on a single patient, for which the processes of creating the model, cutting and observing the effects have been carried out fully and as accurately as possible.

## Results

**Fig. 11:**
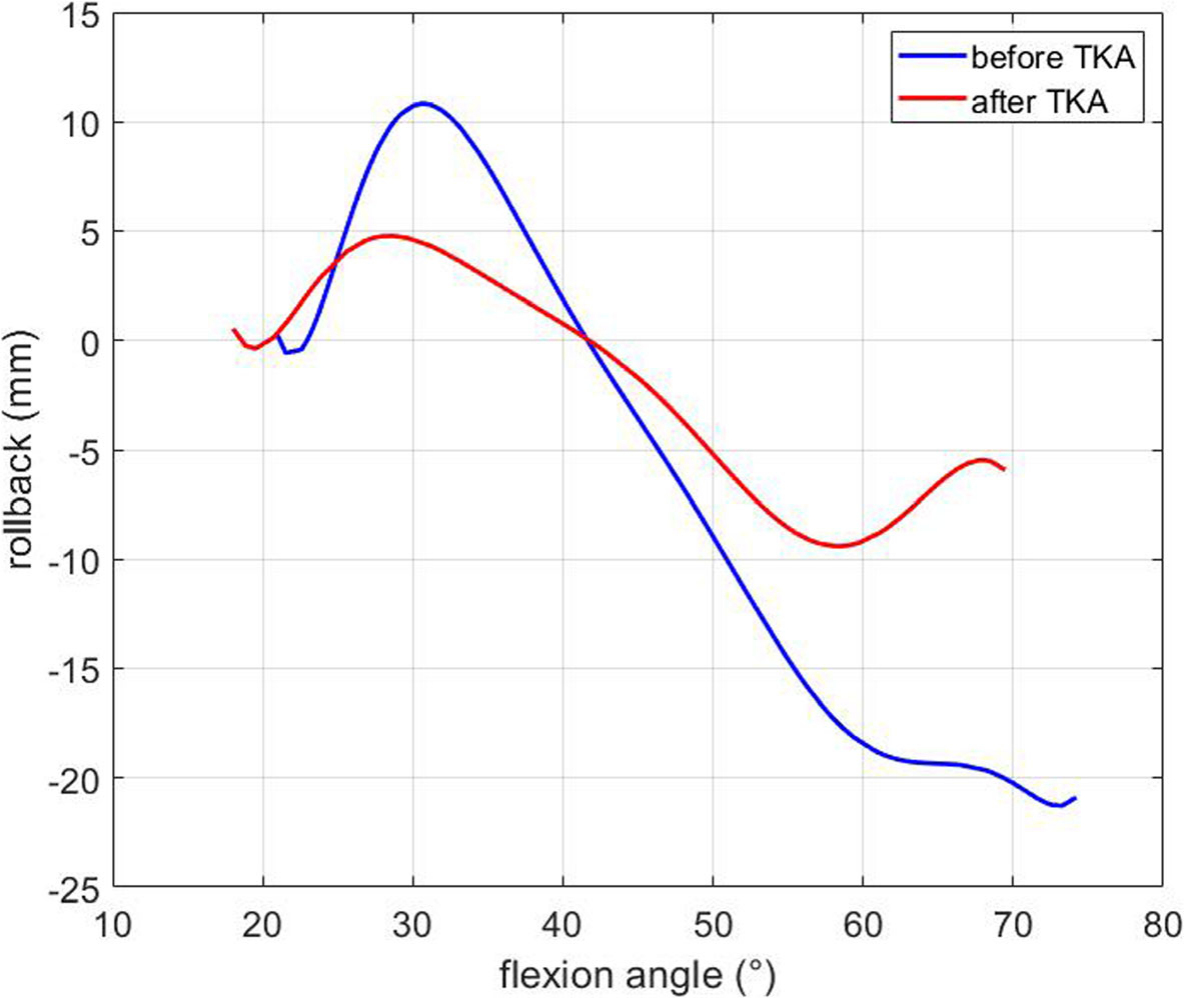
Interpolation of the measured displacement of the contact point between femur and tibia, or rollback, along the horizontal axis

The results are shown in Fig. 11. The shift of the femur condyles towards the back of the tibia plateau is measured from an angle of 20 degrees, which corresponds to the extended position of the model as illustrated in Figs. 9 and 10. The rollback on the preoperative model reaches 10 mm for an angle of about 30 degrees, while the postoperative model only reaches about 5 mm. However, a significant difference can be observed between the position of this contact point before and after the operation, for the same bone and the same flexion. This experiment shows that the prosthesis leads to a change in the femoral rollback. The geometrical change is clearly visible while comparing Figs. 9 and 10. There can be many reasons for this change, such as the position of the prosthesis, but more likely, this modification is due to the shape of the prosthesis.

The rollback curve is flattened after TKA. The knee movement has therefore a smaller amplitude and the overall motion is limited due to the reduction of the femoral rollback. The guided flexion through the four-bar mechanism limits the parameters for the experiment. The focus of this experiment was only on the geom- etry of the femur condyles. The geometry and position of the implant are therefore important parameters that modify the kinematics of the knee and influence the rollback.

## Discussion

Modern knee arthroplasty faces an almost constant proportion of 20% of permanently dissatisfied patients. Doctors claim that multifactorial reasons are responsible [7]. This patient dissatisfaction mainly attributes to a conflict or mismatching between the pre-existing biomechanical characteristics of the knee joint and the new situation created by the prosthesis implantation [2].

In general, improved results depend on a better understanding of the biomechanical situation. In this context, numerous kinematic concepts are currently being used and put up for discussion [9, 12, 14]. Thereby, the positioning of the components and especially the femoral component, play an important role [2, 11, 21]. Conventional implantation concepts (MA, AA, MR) compete with newer and currently popular approaches (KA, RKA, GBA) but the results are controversial. The discussion about all different alignment concepts is endless, including assistance through navigation, robotics, and patient-specific instrumentation [3, 8]. There is no current tool to validate the surgeons’s plan and implantation technique.

In parallel to the clinical studies, modern techniques such as 3D reconstruction, simulation, high-speed cameras, gait analyzes, etc. are increasingly used to analyze the separate biomechanical concepts and thereby to explain the differences in the results [4, 6, 15–17, 20, 22, 27]. These studies can make basic statements about the biomechanical characteristics of the unique alignment concepts. Still, within the different biomechanical concepts, there is always a wide range of results, for which the reason is not known. The usual postoperative follow-up diagnostics cannot detect small differences and incorrect positioning of the implants. But minimal deviations in implant positioning have a significant influence on the objective and subjective result [31, 34]. More generally, there is a lack of techniques to detect the reasons for malfunction after knee arthroplasty, and there is no test evaluating preoperatively the biomechanical consequences of implant positioning after revision with exchange of implants.

In this research, results similar to those of Koh et al. [19] were found, showing that TKA reduces rollback. In this study, the model allows analysis on the knee model of a real patient, thus reducing possible implantation errors due to the com- puterization of the surgery. This study also highlights two key considerations. The first is that currently, 3D printing technology can be combined with the design of mechanisms to replicate parts of the human body, especially the knee joint. The second aspect is that, for a given trajectory and geometry of a knee requiring TKA, the operation on the model provides an opportunity to show the effects of changing the surface of the femur. The prosthesis placement affects the kinematics of the knee definitely because of the change in the curvated femur design, which no longer corresponds exactly to the natural bone shape. Even custom-made implants do not match nature and are so far not superior to standard prefixed implants [2]. Also, in the coronal plane, the changes in the surface design depending on the alignment concept due to the prosthesis implantation could cause a lift-off effect of the medial or lateral femur condyle or could cause hypertension of the ligaments. This experiment clearly shows that the actual implantation method causes significant changes that could be the reason for the kinematic conflicts. These conflicts may be the leading cause of pain. With this model, pain or malfunction can be preoperatively detected and avoided. This model allows the surgeon to have a 3D vision of the problem and to practice on an exact reproduction of the knee geometry of the patient. The presented model arrangement and 3D printing technology combine in a realistic, individualized representation both the shape of a knee joint and its axes of movement. The procedure creates a true-to-life articulated model based on a specific individual case. Moreover, the results of before and after TKA can be simultaneously compared. The 3D printing technology makes the number of possible experiments practically endless and the operation can be performed several times by modifying other parameters if necessary.

In future work, it would be interesting to investigate further the impact of the different alignment concepts on the knee kinematics to improve the understanding of the influence of the prosthesis position on the knee movements in specific patient conditions. The movement of the knee fixed and controlled by the four-bar mechanism, is not reproducible during the operation. The sacrifice of the anterior cruciate ligament and often also of the posterior cruciate ligament during artificial knee implantation causes a tremendous change in biomechanics. Also, this model allows us to compare the preoperative mechanical conditions with the postoperative result and can, therefore, help to address postoperatively implantation related problems. This model provides an additional comparison tool from a different point of view as current methods. This novel method is promising and may provide answers to surgeons about the reasons for the high revision rates of knee replacement surgery. Also, it might be a helpful tool to plan and realize successful revisions.

## Conclusion

In conclusion, this research provides a different perspective of TKA by simulating the knee biomechanics before the operation and after implantation using a 3D printed mechanical model based on the real anatomy of the knee. The patient’s knee movement is directly measured with an infrared camera to create a model with a flexion constrained by the mechanism. The model is ligament-free, which allows the surgeon to operate and implant the prosthesis by following his own TKA technique according to the planned surgical procedure for the patient. The experiments on a model of a real patient’s knee show a reduction of the rollback after surgery. Therefore, TKA modifies the kinematics of the joint and these changes could be the reason for the patient’s dissatisfaction. With this 3D printed model, details in knee movements like axis reconstruction, rotation, component lift-off, ligament hy- pertension, as well as the rollback variations between the preoperative model and the postoperative model can be observed. In an extended future study, it would be interesting to test a series of different knees, different component alignments or to compare the results of different surgical techniques to improve the comprehension of the general results of the TKA, where the technical aspects remain widely discussed.


**Additional file 1**

## Data Availability

The datasets used and/or analysed during the current study are available from the corresponding author on reasonable request.

## Abbreviations

AA: Anatomical alignment
AAA: Adjusted anatomical alignment
GBA: Gap balanced alignment
KA: Kinematic alignment
MA: Mechanical alignment
MR: Measured resection
PSI: Patient-specific instrumentation
RKA: Restricted kinematic alignment
TKA: Total Knee Arthroplasty
